# Physiological and Pathological Changes in the Alveolar Tissues, from Operations for Correction of Irregularities of Teeth

**Published:** 1894-03

**Authors:** J. N. Farrar


### PHYSIOLOGICAL AND PATHOLOGICAL CHANGES IN THE ALVEOLAR TISSUES, FROM OPERATIONS FOR CORRECTION OF IRREGULARITIES OF TEETH.

BY DR. J. N. FARRAR.

*Theory.*—Teeth are moved through absorption of the socket-tissue, or by bending of the alveolar ridge, or both. This absorption or this bending may take place by the tissue-alterations being carried on within their physiological functions, or they may take place outside of these healthy functions and in the pathological. If the tissue-changes can be conducted within the physiological functions, the operation of moving a tooth will be painless. But if the changes are pushed beyond this condition, and into the pathological, the operation will be more or less painful.[1]

Any regulating mechanisms that can be controlled so as to confine the tissue-changes within the healthy line are therefore superior to those that cannot be so controlled. Mechanisms that operate by elastic rubber, or by metallic springs, if the power be not so great as to force the changes to exceed healthy action, will be comparatively painless, but to so control elastic materials that they will not cause pain is difficult, and generally impossible.

This is not the case when mechanisms operated by screws can be used, because by the screw exactness of action can be secured, and (exactly) the proper degree of force can be given. Especially is this true when the management of the mechanism is left to the patient, who knows better than the operator when the greatest degree of force can be given without causing pain.

When these facts are clearly understood and are carried out by a skilful operator, the prejudices of patients against regulating operations will be overcome, and reputation for high skill will begin. This desideration is assured by the screw making it possible to take advantage of the "Law of Labor and Rest," which applies to these tissue actions as well as to other tissue energies.

---

[1] Before Dr. Farrar began to publish (in 1875–76) the results of his investigations in tissue-changes caused by pressure, it was the universal belief that the movement of teeth was "only possible through pathological difficulties."

